# Associations of changes in smoking-related practices with quit attempt and smoking consumption during the COVID-19 pandemic: A mixed-methods study

**DOI:** 10.18332/tid/156454

**Published:** 2022-12-23

**Authors:** Yingpei Zeng, Tzu Tsun Luk, Yongda Socrates Wu, Sau Chai Η. Tong, Wai Yin V. Lai, Tai Hing Lam, Man Ping Wang

**Affiliations:** 1 School of Nursing, The University of Hong Kong, Hong Kong SAR, China; 2 Hong Kong Council on Smoking and Health, Hong Kong SAR, China; 3 School of Public Health, The University of Hong Kong, Hong Kong SAR, China

**Keywords:** smoking cessation, quit attempt, COVID-19, coronavirus, smoking reduction

## Abstract

**INTRODUCTION:**

How changes in smoking routine due to COVID-19 restrictions (e.g. refraining from smoking outdoors and stockpiling tobacco products) influence smoking behaviors remains understudied. We examined the associations of changes in smoking-related practices with quit attempts and smoking consumption in current smokers using a mixed-methods design.

**METHODS:**

In a community-based telephone survey conducted between the second and third wave of the COVID-19 pandemic in Hong Kong, 659 smokers (87.1% male; 45.2% aged 40–59 years) were asked about quit attempts and changes in cigarette consumption and five smoking-related practices since the COVID-19 outbreak. Logistic regression was used to calculate adjusted odds ratio (AOR), adjusting for sex, age, education level, chronic disease status, heaviness of smoking (HSI), psychological distress (PHQ-4) and perceived danger of COVID-19. A subsample of 34 smokers provided qualitative data through semi-structured interviews for thematic analyses.

**RESULTS:**

Favorable changes in smoking-related practices, including having avoided smoking on the street (prevalence: 58.9%) and reduced going out to buy cigarettes (33.5%), were associated with a quit attempt (AOR: 2.09 to 2.26; p<0.01) and smoking reduction (AOR: 1.76 to 4.97; p<0.05). Avoiding smoking with other smokers (50.5%) was associated with smoking reduction (AOR=1.76; p<0.05) but not quit attempt (AOR=1.26; p>0.05). Unfavorable changes, including having increased smoking at home (25.0%) and stockpiled tobacco products (19.6%), were associated with increased smoking (AOR: 2.84 to 6.20; p<0.05). Low HSI (0–2) was associated with favorable changes (p<0.01), while high HSI score (3–6) was associated with unfavorable changes (p<0.01). Qualitative interviews revealed a double-edged effect of staying at home on smoking consumption and that pandemic precautionary measures (e.g. mask-wearing) reduced outdoor smoking.

**CONCLUSIONS:**

Amid the pandemic, favorable changes in smoking-related practices in smokers were mostly associated with quit attempts and smoking reduction, while unfavorable changes were associated with increased smoking. Smokers with higher nicotine dependence were more negatively impacted.

## INTRODUCTION

The implementation of social-distancing measures to contain the COVID-19 pandemic has disrupted daily life activities, including health and related behaviors. A population-based survey in England found that the COVID-19 lockdown was associated with increased quit attempts and successful quitting^1^. Similar surveys in Germany, Italy and New Zealand showed a net increase in cigarette consumption during the lockdown^2-4^. The mixed results may be partly explained by the differences in the local development of the COVID-19 outbreak, pandemic precautionary measures and timing of surveys. Qualitative studies showed that regular smokers generally increased consumption to cope with mental disturbance, such as stress, anxiety and boredom during lockdowns, while reduced activities (e.g. bar visits) decreased consumption among social smokers^5-7^.

Although many smokers changed their smoking habits due to social distancing^8^, how these changes influenced smoking consumption and quitting remains understudied, especially in places with less restrictive social-distancing policies. Hong Kong is a densely populated city with a low cigarette smoking prevalence (9.5% in 2021)^9^. As smoking is banned in indoor public areas and workplaces, smokers often gather and smoke in ‘smoking hotspots’ – outdoor public places where rubbish bins with ashtrays are available. Since the first wave of COVID-19 in January 2020, the government has implemented social-distancing measures, such as gatherings of not more than 4 people and recommendation of work-from-home practice, but no lockdown^10^. People quickly responded by taking precautionary measures like mask-wearing and hand hygiene^10,11^. We observed a decrease in the volume of smokers gathering at smoking hotspots during the pandemic than before^12^. Data from our Youth Quitline showed that the smoking behaviors of most youth smokers (76%) were affected by pandemic precautionary measures such as mask-wearing and reduced social gatherings with peers^13^.

Smoking is associated with severe COVID-19 symptoms and possibly infection^14–16^, and the World Health Organization has recommended smoking cessation to reduce the impact of COVID-19^17^. Studying how changes in smoking-related practices affect smoking behaviors could help governments and cessation services to understand the unintended impact of social distancing measures and to allocate resources accordingly. We examined the changes in smoking-related practices and their associations with quit attempt and changes in cigarette consumption since the COVID-19 outbreak, among current smokers in Hong Kong.

## METHODS

### Study design

A cross-sectional mixed-methods telephone survey was conducted from 7 May to 30 June in 2020, between Wave 2 (March–April) and Wave 3 (July–September) of the COVID-19 outbreak in Hong Kong. During this period, there were sporadic local and imported COVID-19 positive cases (about <10/day) of COVID-19.

### Sampling methods

We targeted Hong Kong residents who currently smoked tobacco products. Potential respondents were identified and screened from existing cohorts of tobacco smokers who formerly joined our territorywide, community-based smoking cessation campaigns organized by the Hong Kong Council on Smoking and Health in 2018 (ClinicalTrials.gov; number NCT03565796) and 2019 (NCT03992742). Details of the campaigns have been published elsewhere^18,19^. Briefly, smokers who joined the campaigns received a brief cessation intervention and were followed for 6 months. Those who successfully quit smoking (biochemically verified) were awarded incentives or prizes. To be eligible for the present study, participants needed to be a current smoker (combustible cigarettes, heated tobacco products or electronic cigarettes), aged ≥18 years, and able to communicate in Chinese. A total of 968 potential respondents were successfully contacted, 769 of whom responded to our survey (response rate: 79.4%). After excluding respondents who had quit smoking before the COVID-19 outbreak (n=80) and those who did not respond to questions related to COVID-19 (n=30), we successfully surveyed 659 respondents. Smokers characteristics were similar between our sample and the general population in Hong Kong: demographic characteristics (male: 87.1% vs 83.1%; aged 40–59 years: 45.2% vs 48.1%, respectively) and daily cigarette consumption (12.1 vs 12.7, respectively)^9^.

### Data collection

All respondents provided verbal informed consent before completing the questionnaire via telephone interview. Respondents were asked if they had changed several smoking-related practices since the outbreak with responses of ‘Yes’ and ‘No’. These included favorable changes (having avoided smoking on the street, avoided smoking with other smokers and reduced going out to buy cigarettes) and unfavorable changes (having increased smoking at home and stockpiled tobacco products). Quit attempts were assessed by asking: ‘Have you ever made any quit attempt since the COVID-19 outbreak?’ with responses of ‘Yes’ and ‘No’. Among respondents who had made quit attempts, we further asked about the methods of quitting (e.g. drinking water/eating snacks or nicotine replacement therapy). Change in cigarette consumption was assessed by asking: ‘Did you change cigarette consumption since the outbreak?’ with responses of ‘No change’, ‘Reduced’ and ‘Increased’, which was used in our previous study^20^.

Sociodemographic information collected included sex, age, education level, employment status and chronic disease history (e.g. hypertension, diabetes and cancer). We assessed daily cigarette consumption and time to the first cigarette of the day, which were used to calculate the heaviness of smoking index (his: 0–6), with higher HSI scores indicating greater nicotine dependence^21^. Psychological distress was assessed by the 4-item Patient Health Questionnaire score (PHQ-4: 0–12), with a score of ≥6 indicating psychological distress^22^. Perceived danger of COVID-19 was measured by asking: ‘From 0 to 10, how dangerous do you think COVID-19 is to your health?’ with higher scores indicating greater perceived danger.

We also conducted individual semi-structured interviews via telephone with a subset of 34 respondents to explain or complement the findings from the survey. A purposive sampling strategy was used to select smokers of different sex and age groups. Following an interview guide, we asked an open-ended question: ‘What has been changed in your smoking habit since the COVID-19 outbreak?’. The interview guide also includes other questions (e.g. misinformation exposure) that were not relevant to the present study’s objective and thus not presented. All interviews were conducted in Cantonese and audio-recorded with the interviewees’ permission. The study endpoint was determined by data saturation. Each interviewee was offered HK$200 (about US$25.5) cash to compensate for their time and effort.

### Statistical analysis

All data analyses were conducted by Stata version 15.1. A two-sided p<0.05 was considered statistically significant. We compared the prevalence of change in smoking-related practices by the respondent characteristics using chi-squared tests. Binary logistic regression was used to calculate the crude (OR) and adjusted odds ratio (AOR) of making a quit attempt by the changes in the five smoking-related practices. Multinomial logistic regression was used to model changes in cigarette consumption (no change as the reference outcome). Apart from sex, age, education level, and chronic disease status, we adjusted for the HSI^23^, psychological distress^20,24^ and perceived danger of COVID-19^25^.

The audio recordings from the qualitative interviews were transcribed verbatim. Two investigators independently analyzed the transcripts by using thematic analyses^26^. Each investigator first read the transcripts to generate an initial thought on the data. Passages related to the research question were coded line-by-line. Codes with similar meanings were clustered into themes. Discrepancies in the coding decision were handled by re-analyses and discussion with a third investigator. All analyses were performed on the original Cantonese. Selected scripts were translated into English for reporting by the bilingual researchers (Cantonese and English).

## RESULTS

Of the 659 respondents, 58.9% (n=388) reported having avoided smoking on the street, 50.5% (n=333) had avoided smoking with other smokers and 33.5% (n=221) reduced going out to buy cigarettes (favorable changes). However, 25.0% (n=165) reported having increased smoking at home and 19.6% (n=129) stockpiled tobacco products (unfavorable changes). The prevalence of participants with favorable changes only was 44.8% (n=292); unfavorable changes only, 7.8% (n=51); both favorable and unfavorable changes, 29.6% (n=193); and neither, 17.8% (n=116).

Table 1 shows the prevalence of changes in smoking-related practices by respondent characteristics. Sociodemographic factors were generally not associated with changes in the five practices with few notable exceptions. More unemployed respondents than employed respondents increased smoking at home (p=0.007). Compared with those without chronic diseases, fewer respondents with chronic diseases avoided smoking on the street (p=0.034) but more stockpiled tobacco products (p=0.007). More respondents with low HSI (0–2) showed favorable changes in all three smoking-related practices (p= 0.001 to <0.001), while more of those with high HSI (3–6) or psychological distress showed all two unfavorable changes (p= 0.003 to <0.001). More respondents with high scores of perceived danger of COVID-19 (≥8) had favorable changes in having avoided smoking on the street (p=0.031) and reduced going out to buy cigarettes (p=0.014), and marginally significant differences in having avoided smoking with other smokers (p=0.062).

**Table 1:** Change in smoking-related practices by participant characteristics in Hong Kong, May–June 2020 (N=659)

*Characteristics*	*Total n (%)*	*Changes in smoking-related practices (row %)*				
		*Avoided smoking on the street (n=388)*	*Avoided smoking with other smokers (n=333)*	*Reduced going out to buy cigarettes (n=221)*	*Increased smoking at home (n=165)*	*Stockpiled tobacco products (n=129)*
**Overall**		58.9	50.5	33.5	25.0	19.6
**Sex**						
Male	574 (87.1)	60.5	52.1	34.2	25.2	19.3
Female	85 (12.9)	60.2	49.4	35.3	27.1	24.7
**Age** (years)						
18–39	246 (39.1)	65.7	58.0	34.3	26.5	19.1
40–59	285 (45.2)	55.6	50.0	33.3	25.0	20.1
≥60	99 (15.7)	58.5	45.4	38.1	20.6	19.8
**Education level**						
Primary or lower	204 (34.2)	54.3	42.6**	35.8	24.8	19.8
Secondary	233 (39.0)	61.7	52.0**	34.2	26.0	21.1
Tertiary	160 (26.8)	61.0	59.1**	35.0	25.6	20.6
**Employment**						
Employed	484 (77.8)	61.5	54.1	32.9	22.5**	18.1
Unemployed	69 (11.1)	52.9	50.0	32.4	39.7**	24.6
Other (students/housekeepers/retired)	69 (11.1)	53.1	40.3	44.1	29.0**	27.9
**Chronic disease status**						
Yes	126 (19.1)	52.0*	45.9	37.1	27.2	28.6**
No	533 (80.9)	62.4*	53.1	33.7	25.0	17.9**
**Heaviness of smoking^a^**						
Mild	330 (56.7)	68.2***	60.6***	39.5**	21.4**	11.9***
Moderate to heavy	252 (43.3)	52.6***	41.3***	26.1**	32.4**	30.2***
**Psychological distress^b^**						
<6	526 (86.5)	60.7	53.4	34.7	22.6***	17.0***
≥6	82 (13.5)	53.8	50.0	30.5	41.5***	36.6***
**Perceived risk of COVID-19^c^**						
<8	285 (43.2)	55.6*	47.5	28.9*	26.0	23.4
≥8	374 (56.8)	64.0*	54.9	38.3*	25.0	17.5

All p values were calculated by chi-squared test.

a Score range 0–6, the score was rated as mild (0–2), moderate (3–4) and heavy (5–6).

b Measured by Patient Health Questionnaire 4 score (range: 0–12), a score ≥6 indicates psychological distress.

c Score range 0–10 with higher scores indicating perceiving higher risk of COVID-19 to health; the score of 8 is the median value in our sample and regarded as the cut-off.

* p<0.05.

** p<0.01.

*** p<0.001.

After the outbreak, 17.6% (n=116) of respondents made a quit attempt. The most common method of quitting was ‘relying on willingness or determination’ (38.8%), followed by ‘no specific method’ (22.4%) and ‘drinking water/gum/snacks’ (15.5%). Only 4.3% (5/116) visited smoking cessation clinics and 3.4% (4/116) used nicotine replacement therapy (Supplementary file Table 1). Table 2 shows that quit attempt was associated with having avoided smoking on the street (AOR=2.09; 95% CI: 1.32–3.31) and reduced going out to buy cigarettes (AOR=2.26; 95% CI: 1.56–3.27), but not with avoiding smoking with other smokers (AOR=1.26; 95% CI: 0.86–1.86). No association between unfavorable changes in smoking-related practices and quit attempt was observed.

**Table 2:** Associations of changes in smoking-related practice with quit attempt since the outbreak in Hong Kong, May–June 2020 (N=659)

*Characteristics*	*Quit attempt n/N (%)*	*Quit attempt*	
		*Crude model OR (95% CI)*	*Adjusted model^a^ AOR (95% CI)*
**Favorable changes**			
**Avoided smoking on the street**			
No (Ref.)	27/251 (10.8)		1
Yes	86/383 (22.5)	2.09 (1.40–3.12)***	2.09 (1.32–3.31)**
**Avoided smoking with other smokers**			
No (Ref.)	50/307 (16.3)		1
Yes	64/329 (19.5)	1.19 (0.85–1.67)	1.26 (0.86–1.86)
**Reduced going out to buy cigarettes**			
No (Ref.)	51/415 (12.3)		1
Yes	62/220 (28.2)	2.29 (1.64–3.20)***	2.26 (1.56–3.27)***
**Unfavorable changes**			
**Increased smoking at home**			
No (Ref.)	87/479 (18.2)		1
Yes	27/161 (16.8)	0.92 (0.62–1.37)	0.99 (0.65–1.48)
**Stockpiled tobacco products**			
No (Ref.)	91/510 (17.8)		1
Yes	21/127 (16.5)	0.93 (0.60–1.43)	1.07 (0.67–1.73)

a AOR: adjusted odds ratio; adjusted for sex, age, education level, chronic disease status, heaviness of smoking, psychological distress and perceived risk of COVID-19.

* p<0.05.

** p<0.01.

*** p<0.001.

For smoking consumption, 445 (67.8%; 95% CI: 64.1–71.4) reported no change, while 172 (26.2%; 95% CI: 22.9–29.8) reduced smoking and 39 (5.9%; 95% CI: 4.3–8.0) increased. Table 3 shows that having avoided smoking on the street and reduced going out to buy cigarettes were associated with smoking reduction (AOR: 1.76 to 4.97; p<0.05), whereas unfavorable changes were associated with increased smoking consumption (AOR: 2.84 to 6.20; p<0.05).

**Table 3:** Associations of changes in smoking-related practice with the changes in cigarette consumption since the outbreak in Hong Kong, May–June 2020 (N=659)

*Characteristics*	*Changes in cigarette consumption n (column %)*			*Reduced vs no change*		*Increased vs no change*	
	*No change*	*Reduced*	*Increased*	*Crude model OR (95% CI)*	*Adjusted model^a^ AOR (95% CI)*	*Crude model OR (95% CI)*	*Adjusted model^a^ AOR (95% CI)*
**Favorable changes**							
**Avoided smoking on the street**							
No (Ref.)	189 (43.8)	46 (27.2)	17 (44.7)		1		1
Yes	243 (56.2)	123 (72.8)	21 (55.3)	2.08 (1.41–3.07)***	1.91 (1.22–3.00)**	0.96 (0.49–1.87)	0.98 (0.44–2.16)
**Avoided smoking with other smokers**							
No (Ref.)	226 (52.3)	63 (37.1)	19 (48.7)		1		1
Yes	206 (47.7)	107 (62.9)	20 (51.3)	1.86 (1.29–2.68)**	1.76 (1.15–2.69)*	1.15 (0.60–2.22)	1.09 (0.51–2.35)
**Reduced going out to buy cigarettes**							
No (Ref.)	332 (76.5)	64 (37.9)	25 (65.8)		1		1
Yes	102 (23.5)	105 (62.1)	13 (34.2)	5.34 (3.64–7.83)***	4.97 (3.20–7.73)***	1.69 (0.84–3.43)	1.93 (0.82–4.53)
**Unfavorable changes**							
**Increased smoking at home**							
No (Ref.)	342 (78.4)	126 (73.7)	13 (33.3)		1		1
Yes	94 (21.6)	45 (26.3)	26 (66.7)	1.30 (0.86–1.96)	1.39 (0.86–2.25)	7.28 (3.60–14.71)***	6.20 (2.78–13.82)***
**Stockpiled tobacco products**							
No (Ref.)	356 (81.7)	134 (79.8)	24 (61.5)		1		1
Yes	80 (18.3)	34 (20.2)	15 (38.5)	1.13 (0.72–1.77)	1.61 (0.95–2.74)	2.78 (1.40–5.54)**	2.84 (1.20–6.71)*

a AOR: adjusted odds ratio; adjusted for sex, age, education level, chronic disease status, heaviness of smoking, psychological distress and perceived risk of COVID-19.

* p<0.05.

** p<0.01.

*** p<0.001.

We interviewed 34 current smokers, including 25 males and 9 females (Table 4). The median time of interviews was about 7 minutes; 10 (29.4%) interviewees made at least a quit attempt and 21 (61.8%) had no change in cigarette consumption. Supplementary file Table 2 shows the individual characteristics. Thematic analyses identified two themes about why smokers changed smoking routines: the impact of staying at home on smoking, and changes in outdoor smoking habits.

**Table 4:** Sample characteristics of the interviewees in Hong Kong, May–June 2020 (N=34)

*Characteristics*	*n (%)*
**Sex**	
Male	25 (74)
Female	9 (26)
**Age** (years)	
18–39	18 (53)
≥40	16 (47)
**Education level**	
Secondary or lower	24 (71)
Tertiary	10 (29)
**Employment status**	
Employment	19 (56)
Unemployment	7 (21)
Retired or housekeepers	8 (23)
**Number of cigarettes per day** (sticks)	
≤10	18 (53)
>11	16 (47)
**Quit attempt**	
Yes	10 (29)
No	24 (71)
**Changes in cigarette consumption**	
No change	21 (62)
Reduced	6 (18)
Increased	7 (20)

*Theme 1: The impact of staying at home on smoking*

Many interviewees reported having increased their duration of staying at home because of working from home and avoidance of outdoor activities, and different impacts on smoking and quitting behaviors. Some smokers reduced smoking consumption because of the presence of children at home (who could not go to schools), or because their family dislike their smoking or the odor of cigarette smoke. A few smokers remained abstained during a prolonged stay at home for 3–4 days:

*‘I smoked less than before because I reduced going out and my children stayed at home.’* (Participant 21, male, 30s)

*‘I didn't want to make my home filled with the smell of tobacco smoke.’* (Participant 7, male, 18 years)

*‘My family don't like me smoking, so I only smoke outside my home like in the workplace … because of the outbreak, sometimes I would be on leave and stay at home for 3 or 4 days. Then, I would stop smoking completely.’* (Participant 25, male, 40s)

However, other smokers increased smoking because of boredom or having nothing else to do as a result of increased spare time from home isolation and reduced social activities:

*‘I reduced going out and stayed at home more often … I want to smoke when I have nothing to do and feel bored and thus smoked more than before.’* (Participant 11, female, 40s)

*‘After the outbreak, I spent more time at home, and my child did not have to go to school. So, I got bored and smoked more (in the kitchen).’* (Participant 17, female, 30s)

*Theme 2: Changes in outdoor smoking habits*

Many smokers gathered in smoking hotspots, which are outdoor places, such as exits of railway stations and entrances of shopping malls. After the outbreak, some interviewees began to avoid clustering with other smokers for fear of getting infected. Some tried to look for places where smokers were less crowded to maintain social distancing, while others stopped smoking outdoors altogether:

*‘When smoking outdoors, I wouldn't go to places crowded with people. If too many people are gathering at the same spot, I would move to other places with more open space to smoke.’* (Participant 25, male, 40s)

After the outbreak, nearly all Hong Kong people voluntarily wear masks in public areas. The risk or inconvenience of mask removal deterred some smokers from smoking outdoors:

*‘Nowadays everyone on the street wears a mask. So, I would not smoke at all when staying outdoors.’* (Participant 13, female, 40s)

## DISCUSSION

In this study, conducted in the early phase of COVID-19 pandemic in Hong Kong, favorable changes in smoking-related practices including having avoided smoking on the street, avoided smoking with other smokers and reduced going out for buying cigarettes, were associated with quit attempt or smoking reduction. Unfavorable changes including having increased smoking at home and stockpiled tobacco products, were associated with increased cigarette consumption. The associations remained significant after adjusting for nicotine dependence (HSI), an established predictor of smoking cessation^23^, as well as psychological stress and perceived danger of COVID-19 as reported in our previous studies^20,25^. Existing studies on COVID-19 and smoking behaviors mostly focused on the role of risk perceptions^27,28^, psychological distress^24^ and lockdown^1-7^. Our findings contribute to the literature by showing how subtle changes in daily smoking routines prompted by the COVID-19 pandemic, changed smoking and quitting behaviors.

We found that changes in smoking-related practices differed among smokers with different characteristics, with nicotine dependence being the most notable factor. More smokers with low nicotine dependence (measured by HSI) had favorable changes in smokingrelated practices, which was associated with smoking reduction and quit attempts. In contrast, more of those with moderate or high nicotine dependence had unfavorable changes, which were associated with increased smoking. These observations, coupled with the inverse association between nicotine dependence and successful quitting^3,23^, suggested that smokers with higher nicotine dependence were more negatively impacted by the pandemic.

Data from qualitative interviews complement the findings from our quantitative analyses by showing how changes in some smoking-related practices led to changes in smoking behaviors. The interviews revealed a mixed effect of home isolation on smoking consumption. Consistent with previous qualitative studies in western countries, boredom or loneliness due to prolonged homestays was a driver of increased smoking in some smokers^5,7^. However, we found that some smokers reduced smoking to protect their children or to avoid conflict with non-smokers in the family. This may be especially salient in Hong Kong because of the crowded living environment and denormalization of smoking, although smoking is legally banned in all public areas. Besides, social distancing and voluntary masking impeded some smokers from smoking in outdoor places. This, coupled with the comprehensive smoke-free policy in public indoor areas in Hong Kong, led to a decrease in smoking in some smokers. Since July 2020 (after our data collection), the Hong Kong government implemented mandatory mask wearing in public places with a penalty. As smokers could not remove their masks for smoking outdoors, further studies are warranted to examine the potential impact on smoking behaviors.

Although about 1 in 6 participants had tried to quit smoking after the onset of the COVID-19 outbreak, most did so without seeking professional help or using evidence-based treatment; only 4.3% had visited a smoking cessation clinic. This was not surprising given that very few smokers (2.0%) reported having used a smoking cessation service according to official data^9^, even though these services are free and effective^29^. Schedule conflicts and low awareness of and interest in cessation treatment are known reasons for the low service usage^29^, but the pandemic had further reduced access to cessation services due to clinic closures and social-distancing behaviors. Leveraging the pandemic as a teachable moment to encourage smoking cessation, greater efforts to promote the accessibility and uptake of smoking cessation service are paramount to reducing smoking prevalence. Mobile health interventions could also be implemented to provide remote cessation support while maintaining social distancing during the pandemic^30,31^.

### Limitations

This study had some limitations. First, the temporality of the associations cannot be determined because of the cross-sectional study design, although it seems more likely that smoking reduction or quit attempts followed changes in smoking practices. Second, smoking and quitting behaviors were self-reported and thus less reliable than biomedical validation which was infeasible because of the telephone survey and the pandemic. Third, unmeasured or residual confounding cannot be excluded in observational studies, but we adjusted for nicotine dependence^23^, as well as factors found to be associated with changes in smoking behaviors during the pandemic, including psychological distress^20-24^ and perceived danger of COVID-19^25^. Fourth, convenience sampling was used to recruit smokers who previously received brief cessation interventions and thus may not be representative of the smokers in the general population. Nevertheless, the similar distribution of sex, age and smoking consumption between our sample and official statistics support the representativeness of our sample^9^. Our observed associations were also likely consistent in smokers with or without prior exposure to minimal cessation support. Lastly, our sample consisted mostly (87.1%) of male smokers, which reflects the male predominance (83.1%) of smoking in Hong Kong^9^ and Eastern culture. This may limit the generalizability of the findings to other places where female smokers are more common.

## CONCLUSIONS

This study found that, during the COVID-19 pandemic, having avoided smoking on the street or with other smokers, and reduced going out to buy cigarettes, were mostly associated with a quit attempt and smoking reduction, whereas having increased smoking at home and stockpiled tobacco products were associated with increased smoking. Smokers with higher nicotine dependence appeared to be more negatively impacted. Smoking cessation interventions need to be strengthened to address smokers with different nicotine dependence and low utilization of proven cessation treatments to promote successful quitting.

## Data Availability

The data supporting this research are available from the corresponding author on reasonable request.
