# Supplementary material for: Associations of changes in smoking-related practices with quit attempt and smoking consumption during the COVID-19 pandemic: A mixed-methods study
Source: Tob Induc Dis. 2022 Dec 23;20:20. doi: 10.18332/tid/156454 (PMC9782258; doi:10.18332/tid/156454)
Supplement: Supplementary file 1 [file TID-20-113-s1.pdf]

Supplementary table 1. The methods of quitting used by those with at least a quit attempt (N=116) in Hong Kong, May–June 2020

	n (%)[a]
Willingness/determination	45 (38.8)
No specific method	26 (22.4)
Drank water/gum/snacks	18 (15.5)
Stopped buying cigarettes	6 (5.2)
Did exercise	5 (4.3)
Visited smoking cessation clinics	5 (4.3)
Nicotine replacement therapy (NRT)	4 (3.4)
Did not specified	7 (6.0)
Others	24 (20.7)

[a] participants can choose more than one method.

Supplementary table 2. Individual characteristics of the interviewees (N=34) in Hong Kong, May–June 2020

Participant number	Sex	Age (years)	Educational attainment	Employment status	Number of cigarettes per day (sticks)[1]	Quit attempt	Changes of cigarette consumption
#1	Male	30-39	Tertiary	Employed	7	Quit for 3 days	No change
#2	Female	40-49	Tertiary	Housekeepers	8	Quit for 6 days	No change
#3	Male	30-39	Tertiary	Unemployed	10	No	No change
#4	Male	40-49	Secondary or below	Employed	15	No	No change
#5	Male	30-39	Secondary or below	Employed	10	No	Reduced
#6	Male	30-39	Secondary or below	Unemployed	20	No	No change
#7	Male	18-29	Secondary or below	Employed	20	No	No change
#8	Male	18-29	Tertiary	Employed	1 can of e-liquid[2]	No	No change
#9	Male	30-39	Secondary or below	Unemployed	15	No	Increased
#10	Male	18-29	Secondary or below	Employed	10	No	Reduced
#11	Female	40-49	Secondary or below	Housekeepers	20	No	Increased
#12	Male	30-39	Tertiary	Employed	20	Quit for 2 days	Increased
#13	Female	40-49	Tertiary	Housekeepers	4	No	Reduced
#14	Male	30-39	Secondary or below	Unemployed	3	No	No change
#15	Female	50-59	Secondary or below	Retired	10	No	No change
#16	Male	18-29	Secondary or below	Employed	20	No	No change
#17	Female	30-39	Secondary or below	Housekeepers	5	Quit for 14 days	No change
#18	Male	40-49	Tertiary	Employed	15	No	No change
#19	Male	40-49	Secondary or below	Employed	15	No	No change
#20	Female	30-39	Secondary or below	Housekeepers	20	No	No change
#21	Male	30-39	Tertiary	Unemployed	10	Quit for 1 day	Reduced
#22	Male	30-39	Tertiary	Employed	10 and 1 can of e-liquid[2]	No	Increased
#23	Female	18-29	Secondary or below	Housekeepers	12	No	No change
#24	Male	40-49	Secondary or below	Employed	8	No	Reduced
#25	Male	40-49	Secondary or below	Employed	10	Quit for 3-4 days	No change
#26	Male	40-49	Tertiary	Unemployed	15	No	No change
#27	Male	50-59	Secondary or below	Employed	20	No	No change
#28	Male	50-59	Secondary or below	Housekeepers	20	Quit for 1 day	No change
#29	Female	50-59	Secondary or below	Employed	10	No	Increased
#30	Male	40-49	Secondary or below	Employed	20	Quit for 1 day	No change
#31	Male	40-49	Secondary or below	Employed	10	Quit for several days	No change
#32	Male	30-39	Secondary or below	Employed	4	Quit for 2-3 days	Reduced
#33	Male	≥60	Secondary or below	Employed	11	No	Increased
#34	Female	30-39	Secondary or below	Unemployed	6	No	Increased

[1] Usually refers to combustible cigarettes
[2] Electronic cigarette users

© 2022 Zeng Y. et al.
